# Comprehensive analysis of differential mRNA, lncRNA and circRNA and their ceRNA network in the Longissimus Dorsi Muscle of Jerseyyak in response to dietary protein levels

**DOI:** 10.3389/fvets.2026.1924842

**Published:** 2026-08-19

**Authors:** Guowu Yang, Bin Ma, Yongfu La, Xiaoming Ma, Xiaoyun Wu, Min Chu, Xian Guo, Lei Wang, Chunnian Liang

**Affiliations:** 1 Key Laboratory of Yak Breeding Engineering of Gansu Province, Lanzhou Institute of Husbandry and Pharmaceutical Sciences of Chinese Academy of Agricultural Sciences, Lanzhou, China; 2 Key Laboratory of Animal Genetics and Breeding on Tibetan Plateau, Ministry of Agriculture and Rural Affairs, Lanzhou, China; 3 Zhangye City Livestock Breeding and Improvement Station, Zhangye, China; 4 Institute of Animal Husbandry and Veterinary Medicine, Tibet Academy of Agriculture and Animal Husbandry Sciences, Lhasa, China

**Keywords:** ceRNA, Jersey-yak, *LASP1*, muscle, whole transcriptome

## Abstract

**Introduction:**

The Jersey-yak, a hybrid offspring of Jersey cattle and yak, exhibits characteristics such as strong adaptability, rapid growth and development, and outstanding production performance. Therefore, Jersey-yaks were fed diets with different crude protein (CP) levels (0%, 15.16%, and 17.90% CP, respectively), and the regulatory mechanisms underlying their growth and development were elucidated.

**Methods:**

In this study, we generated transcriptomic profiles of lncRNAs, circRNAs, miRNAs, and mRNAs from the skeletal muscle tissues of Jersey-yaks, and constructed a ceRNA network using differentially expressed RNAs.

**Results:**

We identified 429, 298, and 84 differentially expressed mRNAs, 394, 356, and 302 differentially expressed lncRNAs, 212, 234, and 20 differentially expressed circRNAs, and 6, 7, and 6 differentially expressed miRNAs in LP vs. MP, LP vs. HP, and MP vs. HP, respectively. These genes were found to be involved in signaling pathways related to skeletal muscle development, cell proliferation, and differentiation, such as the PI3K-Akt, MAPK, HIF-1, Wnt, and Notch signaling pathways. The ceRNA network mainly comprised 6 miRNAs, 6 circRNAs, 8 mRNAs, and multiple lncRNAs. These interactions may affect specific biological processes involved in the growth and development of Jersey-yaks. In addition, association analysis identified genes such as *LASP1*, *DUSP29*, and *PDLIM7*.

**Discussion:**

These findings provide new insights into the molecular mechanisms underlying muscle development in yak and their hybrids.

## 1 Introduction

The yak (*Bos grunniens*) primarily inhabits the Qinghai-Tibet Plateau and surrounding regions at elevations of 3,000–5,500 m, exhibiting remarkable adaptability to harsh environmental conditions such as low temperatures, low oxygen levels, high altitudes, and intense ultraviolet radiation. It is a unique cattle breed resource native to China’s high-altitude, low-oxygen regions (1, 2). China is the primary producer of yak, accounting for over 90% of the global population, primarily distributed across provinces and regions such as Gansu, Qinghai, Sichuan, Xinjiang, Yunnan, and Tibet. Additionally, yak populations are also found in countries such as India, Mongolia, Russia, and Nepal (3). Due to the extremely harsh natural environment in which yaks live, their growth is slow, leading to significant resource wastage. People have begun to crossbreed yak with superior breeds such as Jersey cattle and Angus cattle to improve yak production performance (4, 5). The Jersey-yak crossbreed is the F1 generation resulting from the crossbreeding of Jersey cattle and yak, and it exhibits significant hybrid vigor in terms of production performance. As a crossbred improved offspring of yak, the pre-slaughter live weight, carcass weight, and lean meat percentage of Jersey-yak are significantly higher than those of same-age yak. Research results indicate that under the same feeding conditions, the meat performance of crossbred offspring between Simmental and yak is better, but the meat quality is poorer, while Jersey-yak beef has better color and higher protein content (6). Due to traditional constraints, there has been a lack of innovation in farming techniques and basic infrastructure during the farming process, hindering the large-scale development of the yak industry. Furthermore, the cold weather conditions result in a prolonged dry season of up to 7 months on pastoral grasslands, during which single-pasture grazing cannot meet the nutritional needs of large livestock such as yak and crossbred cattle (7). During the dry season, most livestock do not receive timely and effective supplementary feed, leading to weight loss due to insufficient nutrient intake and increasing pressure on grassland recovery. Therefore, changing livestock feeding patterns during the cold season can not only alleviate pressure on grasslands and forage shortages but also accelerate livestock weight gain and reduce economic losses for herders.

Growth traits are important indicators of the economic value of livestock, primarily including body height, body weight, body length, and growth rate (8). Growth traits are primarily manifested through the growth and development of muscles, with skeletal muscle being an important category within muscles. The regulation of skeletal muscle development is a complex biological process involving multiple stages and various regulatory factors, including transcription factors during myofibrillar differentiation, genes or proteins that regulate muscle growth, and pathways that play a crucial role in the early differentiation of muscle cells and the regulation of protein synthesis (9). In recent years, an increasing number of researchers have utilized transcriptomics technology to explore and elucidate the molecular mechanisms underlying various growth and developmental processes in animals. Dehghanian Reyhan et al. (10) employed whole-transcriptome analysis and competitive endogenous RNA (ceRNA) network analysis to identify molecular regulators involved in intramuscular fat content and fat metabolism across five beef cattle breeds, identifying a total of 34 circRNAs, 57 lncRNAs, 15 miRNAs, and 374 mRNAs. Functional enrichment analysis revealed that these RNAs were enriched in signaling pathways related to fat metabolism, including metabolism, calcium, cGMP-PKG, and thyroid hormone signaling pathways. Additionally, genes such as *MCU*, *CYB5R1*, and *BAG3* were identified as important candidate marker genes for fat metabolism in beef cattle (10). Circular RNAs (circRNAs) play a regulatory role in the development of skeletal muscle in animals. Bao et al. (11) used RNA sequencing to reveal the temporal patterns of circRNA expression at different growth stages in Tibetan sheep and investigated the ceRNA regulatory network in the pectoralis major muscle, elucidating the role of circRNAs in skeletal muscle fiber type conversion and their impact on meat quality, thereby deepening our understanding of the role of circRNAs in Tibetan sheep muscle development.

Skeletal muscle is the primary site for energy conversion, where mechanical energy plays a crucial role in muscle contraction and maintaining muscle function integrity. The growth and development of livestock is essentially a process of tissue and organ growth centered on skeletal muscle, and meat yield and quality directly determine the economic benefits of the livestock industry (12). An analysis of skeletal muscle in Charolais bulls revealed that after a period of protein-free feeding, the reintroduction of protein promotes protein synthesis and reduces muscle breakdown by regulating the mTOR signaling pathway. Mechanistically, *CASTOR2*, which is upregulated during the protein-restricted period, inhibits mTORC1 phosphorylation, whereas *DDIT4*, during the refeeding period, activates the mTOR signaling pathway and promotes protein synthesis (13). Therefore, elucidating the regulatory mechanisms of skeletal muscle growth is a core task in livestock breeding and nutrition research. Research indicates that the growth traits of livestock are directly related to their feeding methods, such as muscle yield, intramuscular fat, and meat quality (14). Many factors influence the quality of animal meat, among which nutrient intake (crude protein) is one of the most important factors. Additionally, as an excellent offspring of the crossbreeding between Jersey cattle and yak, the expression characteristics of lncRNAs and circRNAs in the growth and development process of Jersey-yak have been poorly studied. Therefore, it is necessary to deeply analyze the expression patterns of RNAs (including coding RNAs and non-coding RNAs) during the growth and development of Jersey-yaks fed with different protein levels to fill the gap in the whole transcriptome research. This study utilized RNA sequencing (RNA-seq) technology to investigate the expression characteristics of mRNAs, lncRNAs, and circRNAs in Jersey-yak under different protein-level dietary feeding patterns, providing essential research insights to promote muscle development and enhance productivity in Jersey-yak.

## 2 Materials and methods

### 2.1 Experimental animals and management

This experiment was conducted in Yangnuo Yak Breeding Professional Cooperative in Xiahe County, Gannan Tibetan Autonomous Prefecture. In this experiment, 18 healthy, 6-month-old male Jersey-yaks (bred by artificial insemination of yaks with frozen semen of the same batch of Jersey cattle) were selected. Before the experiment, the animals were weighed and randomly allocated to three treatment groups using R software (v.4.1.2) (4), with six animals per group. All animals were managed under the same grazing conditions. The experimental treatments represented three practical feeding strategies during the cold season: (1) no supplementary feeding (as a control group, low protein level, LP); (2) Supplementary low-protein diet (crude protein content: 15.16%; median protein level, MP); (3) Supplementary high protein diet (crude protein content: 17.90%; high protein level, HP). The specific composition and nutritional level of the two different crude protein level supplemental diets are shown in Supplementary Table S1. The diet formulation was designed in accordance with the NY/T 815–2004 Standard for Beef Cattle Rearing.

It should be noted that the LP treatment did not receive a formulated low-protein supplement. Therefore, LP vs. MP and LP vs. HP comparisons represent the combined effects of supplementary feeding and the associated changes in nutrient supply, whereas the MP vs. HP comparison more directly evaluates the effect of crude protein concentration within supplemented animals.

### 2.2 Weight data collection and sample collection

At the beginning of the formal experiment, 18 Jersey-yaks were weighed and the initial body weight (IBW) of all Jersey-yaks in the three treatment groups was recorded. After the formal test began, all the Jersey-yak in the three groups were weighed on the 20th of each month until the end of the formal test. The weight data recorded during the period were the fasting weight of the Jersey-yak before grazing. Total weight gain (TWG) and average daily gain (ADG) were calculated based on the body weight data recorded during the formal experiment.

At the end of the feeding experiment, 3 Jersey-yaks were randomly selected from each group for slaughter, and the muscle histology of the longissimus dorsi muscle was studied. The samples of the longissimus dorsi muscle tissue from the 12th to 13th ribs of the left half carcass were quickly collected during slaughter, immediately stored in liquid nitrogen, and brought back to the laboratory for transcriptome sequencing and real-time quantitative polymerase chain reaction (RT-qPCR) analysis. All samples are stored in the refrigerator (−80 °C).

### 2.3 RNA isolation, cDNA library construction, and Illumina sequencing

Total RNA was isolated from the LD muscle samples of Jersey-yak using TRIzol reagent (Invitrogen, CA, United States). Then, total RNA was purified using DNase and RNeasy Mini Kit (Qiagen, CA, United States). Using RNA as a template, use the first 6 base random primers and reverse transcriptase to synthesize the first strand of cDNA, and use the first strand cDNA as a template to synthesize the second strand cDNA. The quantity and quality of extracted total RNA were measured using NanoDrop 2000c spectro-photometer (Thermo Fisher Scientific, DE, United States), Bio-Photometer (Eppendorf, Ham-burg, Germany) and 1% agarose gel electrophoresis. Finally, RNA integrity was assessed using the RNA NanoSeq 6,000 Assay Kit for the Agilent 2,100 Bioanalyzer system (Agilent Technologies, CA, United States).

After RNA extraction, purification and library construction, the samples were subjected to Next-Generation Sequencing (NGS) based on Illumina HiSeq (LC Sciences, Houston, TX, United States) sequencing platform. These libraries were then subjected to Paired-end (PE) sequencing.

### 2.4 Data preprocessing and read mapping

The samples were sequenced using a sequencing platform, generating image files. These files were converted using the platform’s built-in software to produce raw FASTQ data. The sequencing data contained some reads with adapters and low-quality reads, which could significantly interfere with subsequent information analysis. Therefore, Cutadapt was used to remove sequences with adapters at the 3′ end and reads with an average quality score below Q20. First, Bowtie2 creates an index of the reference genome, and then the Hisat2 (15) software is used to align the filtered read sequences with the yak reference genome (Bosgru_v3.0) (16). Hisat2 employs an improved BWT algorithm, which offers faster processing speeds and lower resource requirements (17).

### 2.5 LncRNAs acquisition and splicing

LncRNAs refers to long non-coding RNA with a fragment length greater than 200 nt. Based on the structure and non-coding characteristics of LncRNAs, we screened candidate LncRNAs using three strict screening criteria. Subsequent analyses were performed on genes and the strictly screened candidate LncRNAs. First, we used the Stringtie software (18) (v.2.2.0) to assemble transcripts using the alignment results from Hisat2 (15). After removing transcripts with uncertain strand orientation, we screened for LncRNAs in the remaining assembled transcript set. (1) Screen transcripts with a length ≥200 bp and ≥2 exons; (2) Screen transcripts with Class-code x/u/i. Specifically, Anti-sense LncRNAs, Intergenic LncRNAs, and Intronic LncRNAs transcripts. (Here, x refers to transcripts on the opposite strand of the reference transcript that cover its exons; u refers to unknown transcripts in intergenic regions; i refers to transcripts entirely within an intron of the reference transcript); (3) Screen for LncRNAs with coverage >3 in at least one sample. That is, the transcript appears at least three times in one sample. Conduct encoding potential analysis on candidate LncRNAs to determine whether these new transcripts have the ability to encode proteins, thereby further screening the new transcripts to obtain high-confidence LncRNAs. We simultaneously employed the following three methods for analysis: PLEK (v.1.2) (19), CNCI (v.2.0) (20), and Pfamscan (v.27.0) (21). All software uses the default settings. New transcripts deemed by all three software tools to lack coding potential were considered high-confidence LncRNAs and used for subsequent analysis.

### 2.6 CircRNAs and miRNAs identification

First, the raw data is filtered, and the resulting high-quality sequences (Clean Data) are aligned to the reference genome of the species. The data filtering criteria primarily include: (1) using Cutadapt to remove sequences with adapters at the 3′ end; (2) removing reads with an average quality score below Q20. For sequences that did not align, 20 bp are trimmed from both ends to obtain Anchor Reads. These anchor reads are then realigned to the genome, and based on the alignment results, find_circ (v.1.2^1^) is used to identify circular RNAs (22). Next, highly reliable circular RNAs are filtered based on the following criteria: (1) breakpoints = 1; (2) anchor_overlap ≤ 2; (3) edit ≤ 2; (4) n_uniq > 2 and n_uniq > samples and n_uniq > int. (samples/2); (5) best_qual_A > 35 or best_qual_B > 35; (6) the CircRNA length is less than 100 k. We extracted 20-bp regions at both ends of unaligned read sequences from the Hisat2 alignment results as anchor sequences and used Bowtie2 (v.2.3.0) (23) to realign them to the genome to detect circular RNAs. Based on the circRNA identification results from ‘find_circ’, we calculated the “Reads Count” value as the raw expression level of the circRNA, and subsequently standardized the circRNA expression levels using TPM (24).

This project utilized HiSeq single-end sequencing. After sequencing, adapters were removed from the raw sequences, which were then quality-trimmed based on sequence quality by searching the raw sequences in 5-base windows. The number of clean reads with lengths between 18 nt and 36 nt was counted. Using BLAST, the unique reads were aligned against the Rfam13 database to screen for the four known classes of ncRNA: rRNA, tRNA, snRNA, and snoRNA. Reads that mapped to these four classes of ncRNA were discarded. For reads not annotated to the aforementioned four categories of ncRNA, use BLAST to align them against mature miRNA sequences in miRBase22^2^. Screen for conserved miRNAs from these results. Align sequences not mapped to the Rfam or miRBase databases against the yak genome. Use the mireap algorithm to predict new miRNAs. Using the 3’UTR sequences of yak mRNAs as target sequences, perform miRNA target gene prediction for differentially expressed miRNA sequences using miRanda.

### 2.7 Differential expression genes and pathway analysis

We used Stringtie (v.2.2.0) software (18) to perform reads counting statistics on mRNAs and LncRNAs at the transcript level, obtaining the raw expression levels of mRNAs and LncRNAs. We then used Fragments Per Kilo bases per Million fragments (FPKM) (25) to normalize the expression levels and calculate their expression levels. We standardized the counts for each sample using the DESeq software (26) and calculated the fold change (FC). We used the negative binomial distribution test (NB) to assess the statistical significance of the count differences. Finally, differentially expressed mRNAs (DEGs), lncRNAs (DELs), circRNAs (DECs), and miRNAs (DEMs) were identified based on the criteria |log2FC| > 1 and *p* < 0.05. To further understand gene function, functional pathway enrichment analysis was performed using Gene Ontology (GO^3^) terms and the Kyoto Encyclopedia of Genes and Genomes (KEGG^4^).

### 2.8 Construction of ceRNA network

First, using miRanda (v.3.3a) to predict interactions between miRNA-mRNA, miRNA-lncRNA, and miRNA-circRNA. Those with absolute coefficient values greater than 0.8 are considered relevant to network construction, and *p* < 0.05 is deemed statistically significant. Interactions with energy not exceeding −20 kcal/mol were retained, and the network was visualized using Cytoscape (v.3.9.1) software.

### 2.9 Transcriptome-proteome association analysis

Based on previously published TMT proteomics data (27), we analyzed the transcriptomic and proteomic results of the longissimus dorsi muscle from Jersey-yak, performed a correlation analysis of differentially expressed genes (DEGs) and differentially expressed proteins (DEPs), and integrated transcriptomic and proteomic data from the same sample source. Conduct a correlation analysis of DEGs and DEPs using R software (v.4.1.2).

### 2.10 Quantitative real-time PCR for validating gene expression

Randomly selected differentially expressed RNAs were subjected to real-time quantitative PCR (qRT-PCR) experiments. The qRT-PCR analysis was performed using RNA samples from 3 animals per treatment group, representing 3 independent biological replicates. Primers were designed using Primer Premier 5.0 software, with *β-actin* as the internal reference gene (Supplementary Table S9). RNA was reverse transcribed into cDNA using the TransScript First-Strand cDNA Synthesis SuperMix kit. The total reaction volume for qRT-PCR was 20 μL, containing 10 μL SYBR Green Premix Pro Taq HS Qpcr kit (Aikrui Bio, Hunan, China), 0.4 μL each of forward and reverse primers, 1 μL cDNA, and 8.2 μL ddH₂O. The amplification program was set according to the kit instructions, with an annealing temperature of 58 °C and 45 cycles. The relative expression level of RNA was calculated using the 2^-△△CT^ method based on the cycle threshold (CT). Data are presented as the mean ± SE.

### 2.11 Statistical analysis

Excel was used to summarize the body weight data, and then SPSS25.0 software (IBM, Armonk, NY, United States) was used to perform One-Way ANOVA analysis of variance and LSD multiple comparisons on all data. The results were expressed as mean ± SEM. *p* < 0.05 indicated significant difference.

## 3 Results

### 3.1 Effects of different protein levels on body weight of Jersey-yak

The effect of crude protein level in the supplementary diet on the body weight of Jersey-yak is shown in Table 1 (27). The results showed that there was no significant difference in body weight between the initial test and the 30th day (*p* > 0.05). There were significant differences in body weight at 60 days, 92 days and 120 days (*p* < 0.05). The results showed that the body weight of MP group and HP group was significantly higher than that of LP group from the 60th day after grazing (*p* < 0.05). ADG and TWG in MP group and HP group were significantly higher than those in LP group (*p* < 0.05), and there was no significant difference in ADG and TWG between MP group and HP group (*p* > 0.05). The results showed that after supplementary feeding after grazing, the growth performance of Jersey-yak was significantly improved from 60 days.

**Table 1:** Effects of dietary levels on body weight of Jersey-yak.

Items (kg)	Treatment group (unit: kg, Mean ± SEM^1^)			*p*-value
	LP (*n* = 6)	MP (*n* = 6)	HP (*n* = 6)	
Pre-test weight (kg)	64.92 ± 4.73	63.84 ± 4.54	64.84 ± 5.91	0.986
Initial weight	65.80 ± 4.89	67.08 ± 5.05	70.36 ± 4.78	0.798
30 days weight	68.88 ± 3.40	74.28 ± 4.64	82.32 ± 5.03	0.155
60 days weight	72.40 ± 4.05^b^	83.16 ± 4.35^ab^	88.44 ± 3.83^a^	0.046
90 days weight	75.34 ± 4.47^b^	88.66 ± 4.67^a^	94.10 ± 3.01^a^	0.020
120 days weight	73.72 ± 4.76^b^	91.88 ± 5.19^a^	96.76 ± 3.17^a^	0.008
ADG	0.07 ± 0.05^b^	0.21 ± 0.05^a^	0.22 ± 0.03^a^	0.027
TWG	7.92 ± 5.60^b^	24.80 ± 6.04^a^	26.40 ± 3.57^a^	0.027

Data are expressed as mean ± SEM. LP, low protein level; MP, median protein level; HP, high protein level. ADG, average daily gain; TWG, Total weight gain. Different letters on the shoulder markers in the same row of data indicate a significant difference (*p* < 0.05). This table is adapted from a previously published article (27), which is licensed under a Creative Commons Attribution 4.0 International License (CCBY 4.0).

### 3.2 Analysis of RNA-seq data

In order to evaluate the genes related to the growth and development of Jersey-yak, skeletal muscle tissues were collected and all mRNA and non-coding RNAs were analyzed by high-throughput sequencing. After screening out low-quality and redundant readings, we obtained an average of 74,483,798, 72,003,778 and 76,062,278 clean readings from the skeletal muscle samples of the three groups of LP, MP, and HP, respectively. Among them, Q30 was greater than 93.69%, and the successful comparison of the readings with the reference genome was about 93.57% (Supplementary Table S2). We detected a large number of mRNAs, lncRNAs and circRNAs (Supplementary Table S3). It was found that the length of lncRNAs was mainly concentrated at 200 bp. The proportion of lncRNAs containing two exons accounted for the majority, while most of the mRNAs had more than 10 exons. The average length of circRNAs detected was 1925 bp, and 10.48% of them were longer than 2000 bp. The length distribution of circRNAs in all samples was relatively uniform. The sequencing quality was stable across all samples, with no significant batch-to-batch variation.

### 3.3 Differentially expressed mRNAs during growth and development of Jersey-yak

**Figure 1:**
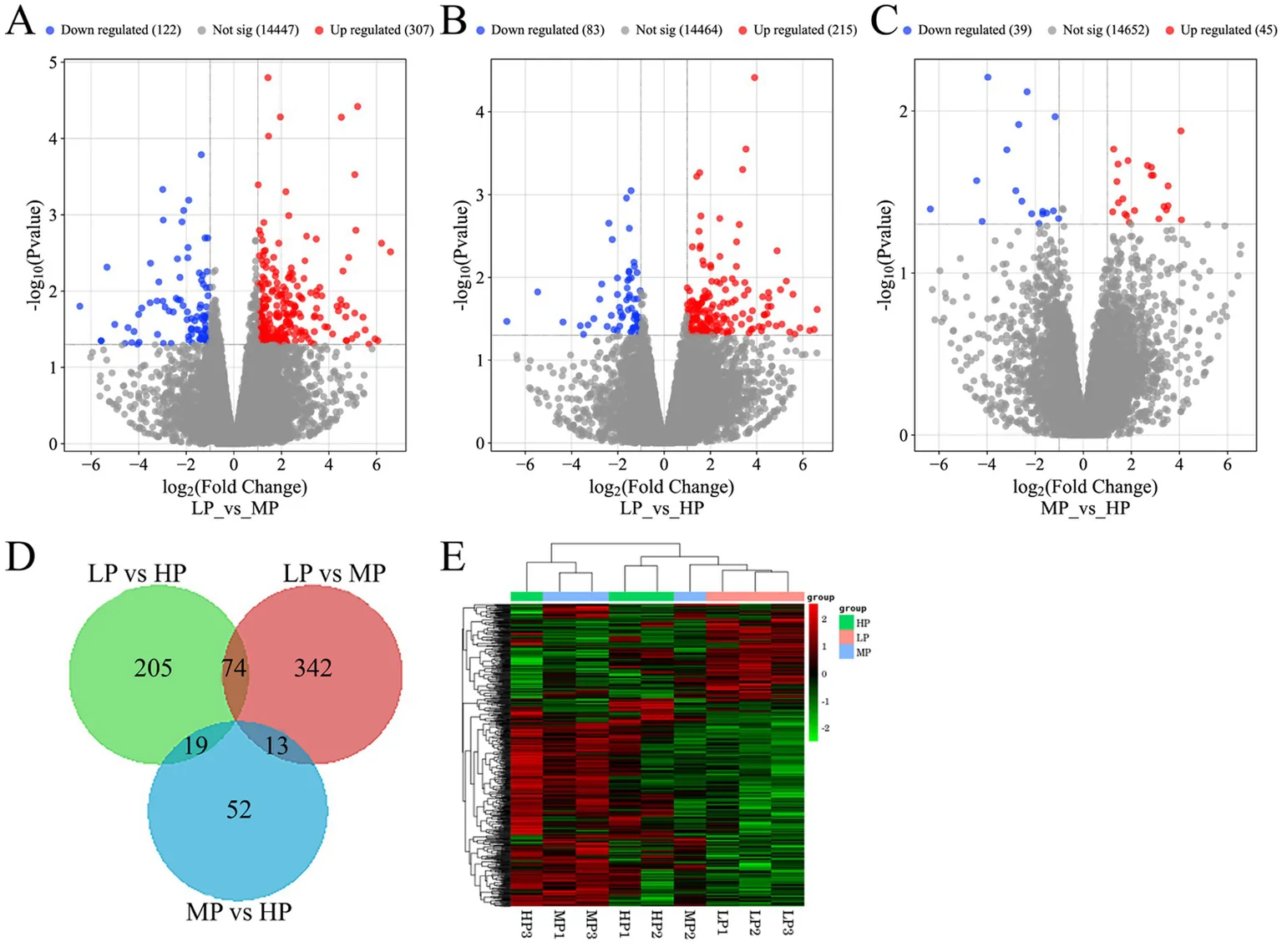
Comparative analysis of differentially expressed mRNAs among different groups of Jersey-yaks. **(A)** Volcano plot showing the differential expression of mRNAs in LP vs. MP. **(B)** Volcano plot showing the differential expression of mRNAs in LP vs. HP. **(C)** Volcano plot showing the differential expression of mRNAs in MP vs. HP. **(D)** The co-expressed DEGs in three comparison groups. **(E)** Heatmap showing the cascading relationship of DEGs in different comparison groups. The colors range from green to red, indicating expression levels from low to high.

We used DESeq to analyze the differential expression of mRNAs. A total of 429, 298 and 84 mRNAs were identified to be significantly differentially expressed in LP vs. MP, LP vs. HP and MP vs. HP groups, respectively (Supplementary Table S4). Among these, in the LP vs. MP comparison group, 122 were downregulated and 307 were upregulated (Figure 1A). In the LP vs. HP comparison group, 83 were downregulated and 215 were upregulated (Figure 1B). In the MP vs. HP comparison group, 39 were downregulated and 45 were upregulated (Figure 1C). These genes include phosphoglycerate kinase 1 (*PGK1*), myogenin (*MYOG*), cellular communication network factor 1 (*CCN1*), enolase 1 (*ENO1*), insulin-like growth factor binding protein 5 (*IGFBP5*), and insulin-like growth factor binding protein 6 (*IGFBP6*), which may be associated with muscle growth, fat deposition, and regulation of growth and development. Using a Venn diagram to highlight DEGs associated with each pair of samples (Figure 1D), 74 differentially expressed mRNAs were found to be shared between the LP vs. HP and LP vs. MP comparison groups. Cluster analysis indicated high reproducibility among DEGs across the three groups while revealing substantial differences between the three groups (Figure 1E).

**Figure 2:**
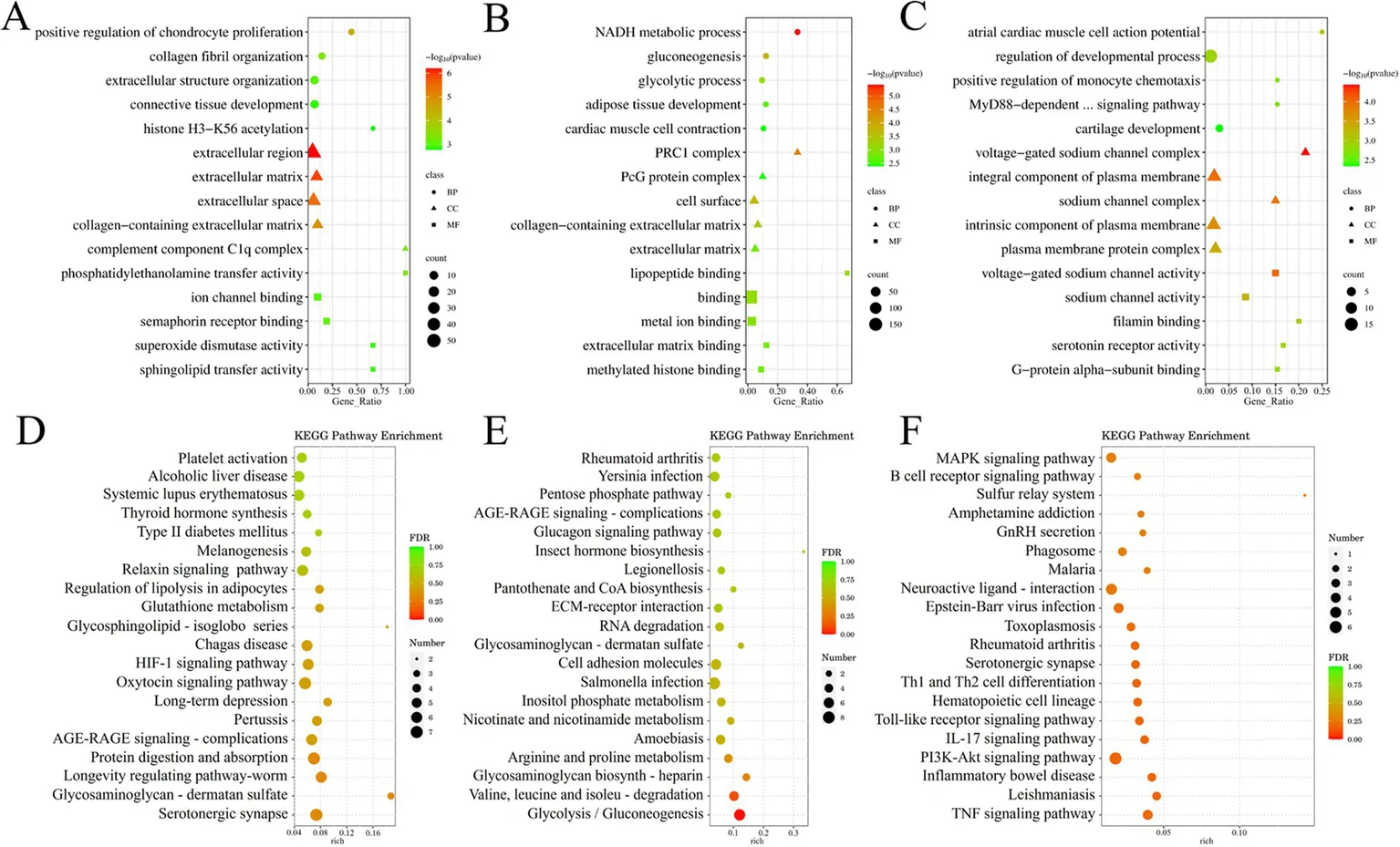
Functional enrichment analysis of DEGs. GO enrichment analysis of LP vs. MP, LP vs. HP, and MP vs. HP **(A–C)**. KEGG enrichment analysis of LP vs. MP, LP vs. HP, and MP vs. HP **(D–F)**. The general value range of FDR is 0–1, and the closer to zero, the more significant the enrichment.

To gain a deeper understanding of the biological functions of DEGs, we performed GO and KEGG analyses (Figure 2 and Supplementary Table S5). The results showed that DEGs in the LP vs. MP group were significantly enriched in positive regulation of chondrocyte proliferation, collagen fibril organization, extracellular structure organization, connective tissue development, histone H3-K56 acetylation, and other biological processes at the cellular and tissue levels that are crucial for the regulation and formation of growth and development in Jersey-yak. They also participate in pathways involved in important physiological processes such as protein digestion and absorption, the HIF-1 signaling pathway, and the regulation of lipolysis in adipocytes, which are related to protein digestion, energy metabolism, and fat metabolism (Figures 2A,D). In the LP vs. HP group, the DEGs were significantly enriched in biological process entries such as NADH metabolic process, gluconeogenesis, glycolytic process, and cardiac muscle cell contraction, which play crucial roles in the growth and development of Jersey-yak. Additionally, they were enriched in biological process entries related to fat metabolism, such as adipose tissue development (adipose tissue development). They are also significantly enriched in cellular components of protein complexes with important functions in regulating gene expression, such as the PRC1 complex and the PcG Protein Complex. Furthermore, it is involved in the regulation of biological processes such as energy metabolism, nitrogen metabolism, cellular physiological functions, and growth and development processes. This includes signaling pathways and metabolic pathways such as ECM-receptor interaction, glycolysis/gluconeogenesis, glucagon signaling pathway (Figures 2B,E). Finally, the DEGs in the MPvsHP group were significantly enriched in biological process entries related to the regulation of developmental processes, cartilage development, and other processes crucial for maintaining the body’s immune defense, resisting pathogens, and maintaining immune balance, such as the MyD88-dependent toll-like receptor signaling pathway. They were also involved in pathways such as the PI3K-Akt signaling pathway, the MAPK signaling pathway, and the Toll-like receptor signaling pathway (Figures 2C,F).

### 3.4 Total lncRNA and differentially expressed lncRNA during the growth and development of Jersey-yak

**Figure 3:**
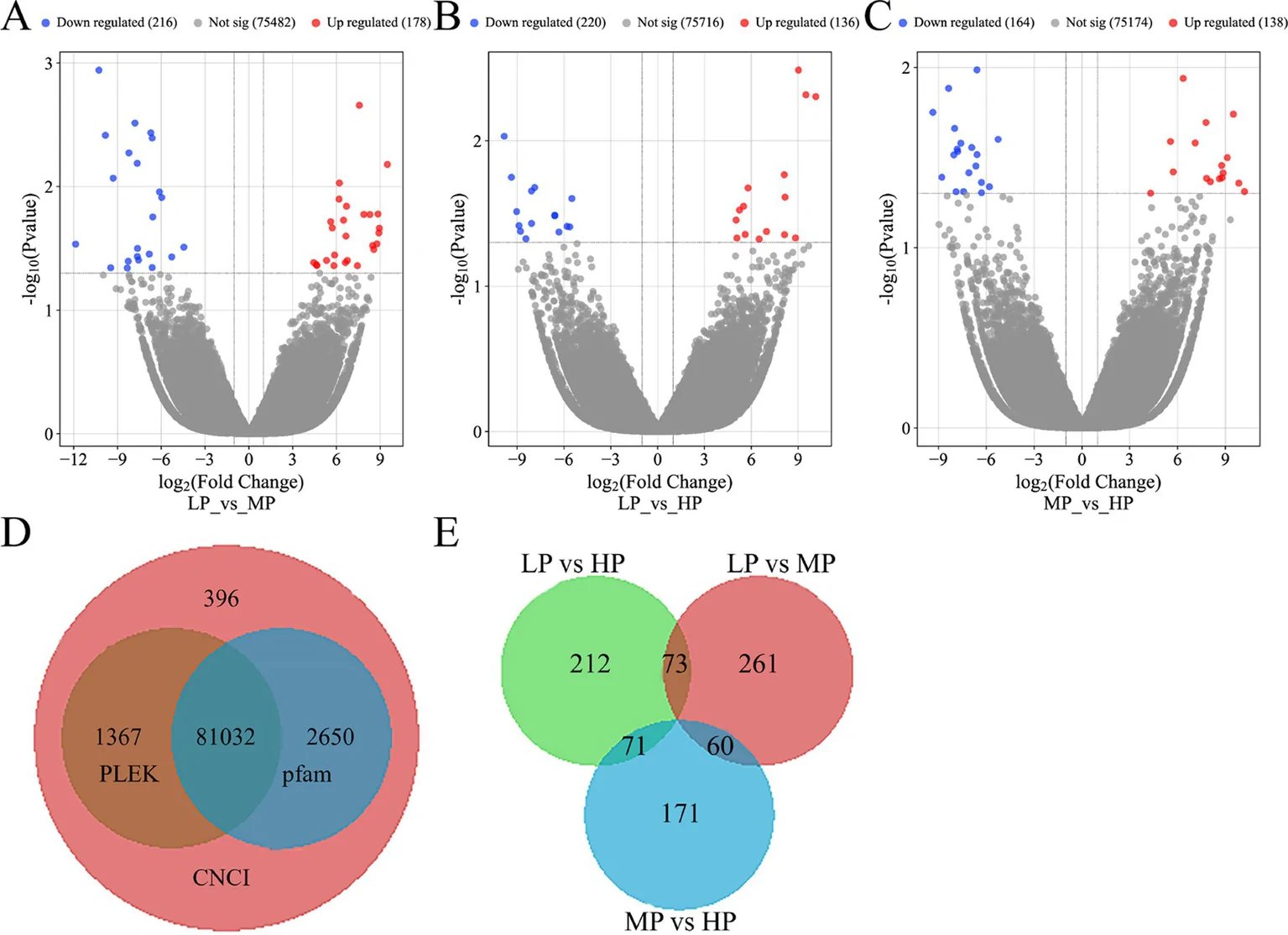
Comparative analysis of differentially expressed lncRNAs among different groups of Jersey-yaks. **(A)** Volcano plot showing the differential expression of lncRNAs in LP vs. MP. **(B)** Volcano plot showing the differential expression of lncRNAs in LP vs. HP. **(C)** Volcano plot showing the differential expression of lncRNAs in MP vs. HP. **(D)** The number of lncRNAs detected by CNCI, pfam, and PLEK software in muscle tissue. **(E)** The co-expressed DELs in three comparison groups.

CNCI, PLEK and Pfam methods were used for comparative analysis to identify lncRNAs in muscle tissue RNA samples, resulting in a total of 81,032 lncRNAs detected in tissues (Figure 3D). Differential expression analysis showed that 394, 356 and 302 lncRNAs were significantly differentially expressed in the three comparison groups of LP vs. MP, LP vs. HP and MP vs. HP, respectively. In the comparison group LP vs. MP, 178 DELs were significantly upregulated and 216 DELs were significantly downregulated (Figure 3A); in the comparison group LP vs. HP, 136 DELs were significantly upregulated and 220 DELs were significantly downregulated (Figure 3B); in the comparison group MP vs. HP, 138 DELs were significantly upregulated and 164 DELs were significantly downregulated (Figure 3C). Through the Wayne diagram to highlight the DELs associated with each pair of samples, it was found that 73 DELs were co-expressed between the LP vs. HP and LP vs. MP comparison groups (Figure 3E). In order to reveal the potential function of identified lncRNAs in the muscle growth and development of Jersey-yak, we predicted the target genes of lncRNAs (Supplementary Table S6).

**Figure 4:**
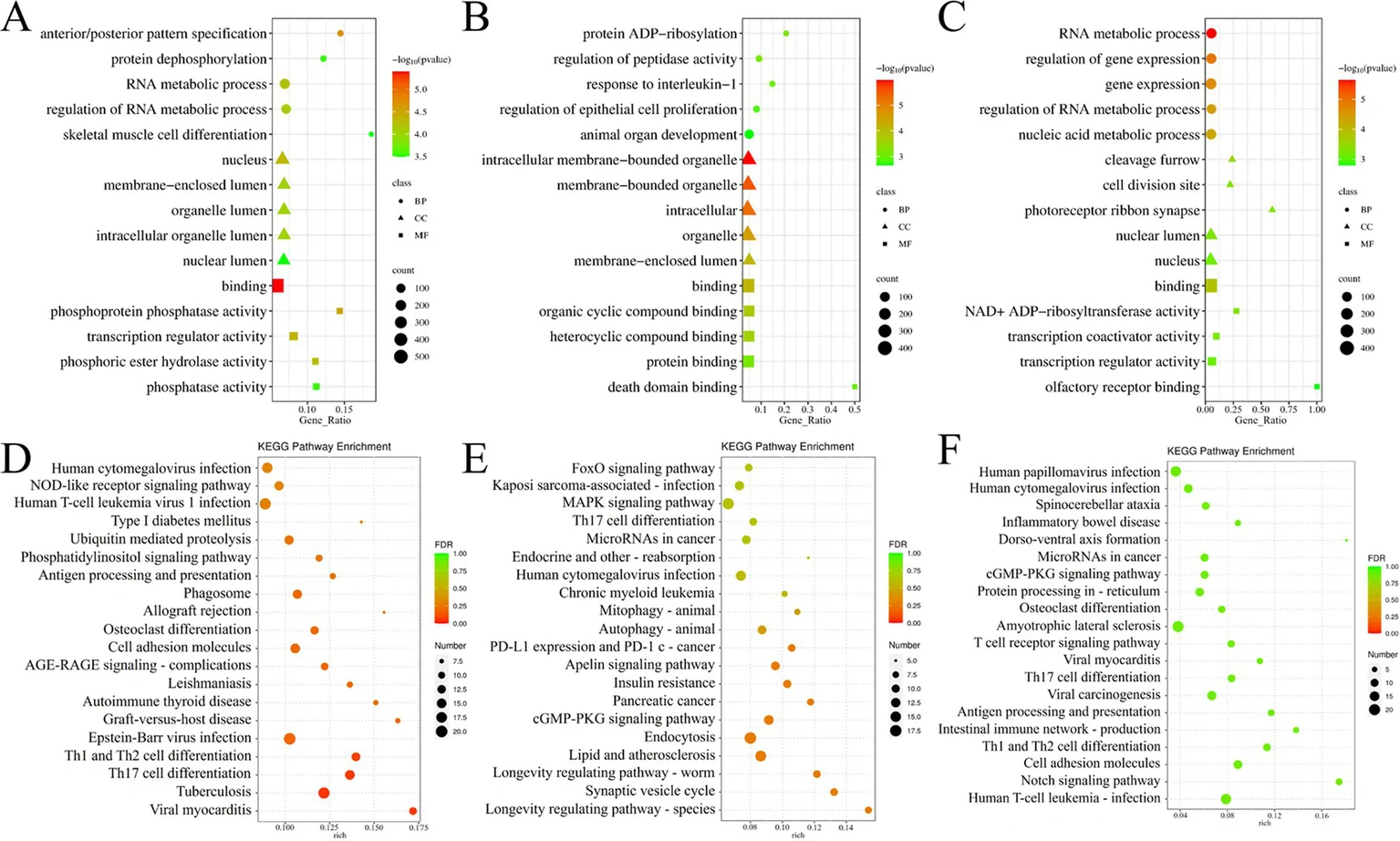
Functional enrichment analysis of DELs. GO enrichment analysis of LP vs. MP, LP vs. HP, and MP vs. HP **(A–C)**. KEGG enrichment analysis of LP vs. MP, LP vs. HP, and MP vs. HP **(D–F)**. The general value range of FDR is 0–1, and the closer to zero, the more significant the enrichment.

The GO enrichment analysis and KEGG pathway analysis revealed several important pathways associated with muscle tissue development in the three comparison groups (Figure 4 and Supplementary Table S6), such as protein dephosphorylation, skeletal muscle cell differentiation, protein ADP-ribosylation, regulation of epithelial cell proliferation, regulation of peptidase activity, animal organ development, cGMP-PKG, MAPK, and Wnt signaling pathway. These pathways play a key role in cell proliferation and differentiation, as well as skeletal development. It was found that the target genes of cis-acting lncRNAs were significantly enriched in the Notch signaling pathway in the MP vs. HP group. This pathway is a highly conserved intercellular signaling pathway that plays an important role in animal development, angiogenesis, skeletal development, cell fate determination, and tissue repair (28, 29).

### 3.5 Differential expression of miRNAs and circRNAs during growth and development of Jersey-yak

In the LP vs. MP, LP vs. HP, and MP vs. HP samples, there were fewer DEMs, with only 6 (5 upregulated, 1 downregulated), 7 (6 upregulated, 1 downregulated), and 6 (1 upregulated, 5 downregulated) DEMs, respectively (Supplementary Table S4). GO functional enrichment analysis revealed that the target genes of these DEMs were significantly enriched in biological processes related to promoting cellular function and the animal’s normal development, repair, and adaptation to the environment, such as positive regulation of cellular process and positive regulation of macromolecule biosynthetic process (Supplementary Table S7). KEGG pathway analysis revealed that the target genes of these DEMs were significantly enriched in pathways critical for animal growth and development, such as the Apelin, PI3K-Akt, AMPK, ECM-receptor interaction, Wnt, Hippo, Ras, and MAPK signaling pathway. Among these, the Hippo, MAPK, and AMPK signaling pathway are associated with lipid metabolism in animals (Supplementary Table S7).

**Figure 5:**
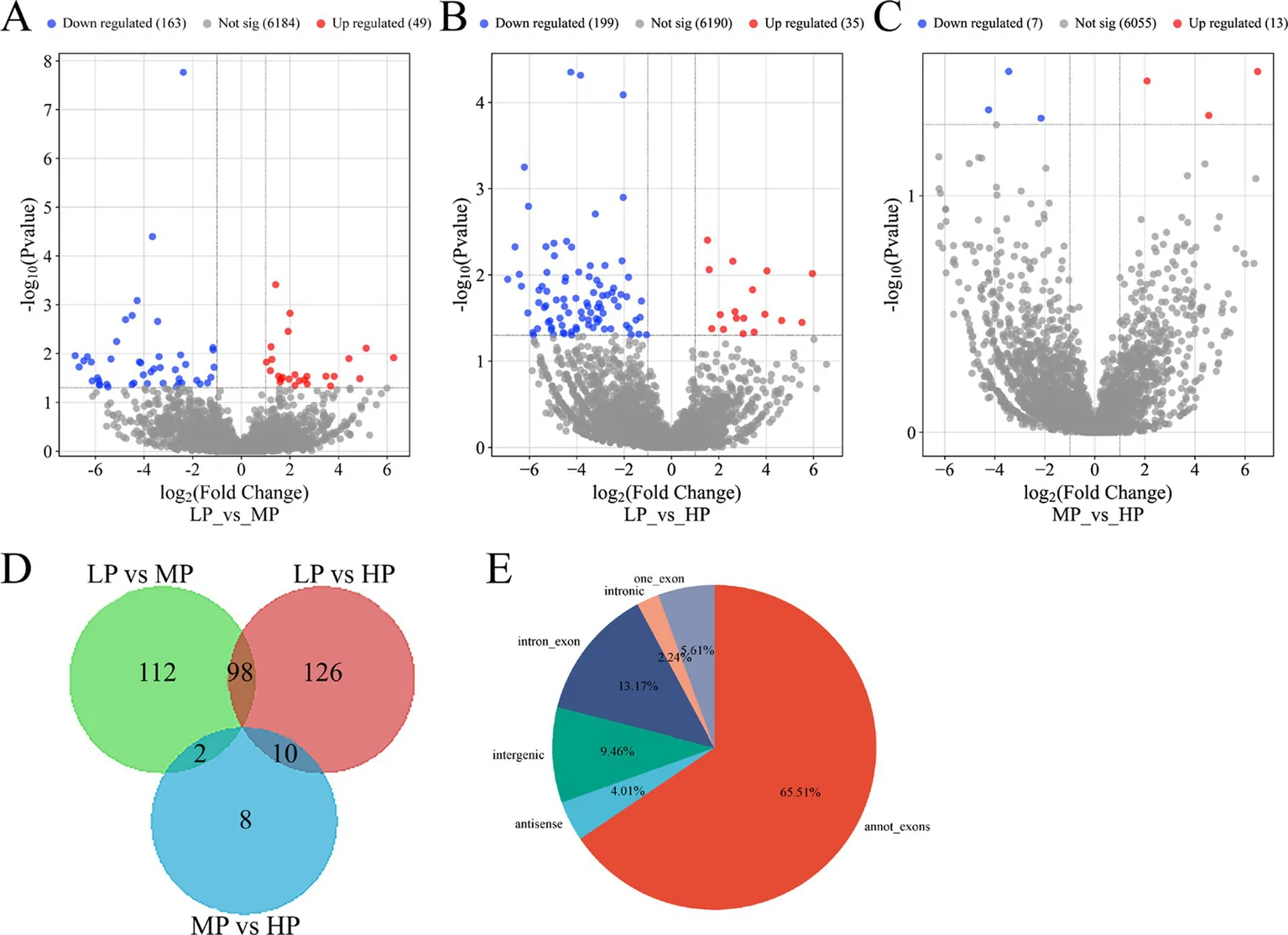
Comparative analysis of differentially expressed circRNAs among different groups of Jersey-yaks. **(A)** Volcano plot showing the differential expression of circRNAs in LP vs. MP. **(B)** Volcano plot showing the differential expression of circRNAs in LP vs. HP. **(C)** Volcano plot showing the differential expression of circRNAs in MP vs. HP. **(D)** The co-expressed DECs in three comparison groups. **(E)** Distribution map of circRNAs structural types.

Among the circRNAs annotated in this study, exon-containing circRNAs accounted for 71.12%, including multiple exon circRNAs (annot) and single exon circRNAs (one), which accounted for 65.51 and 5.61%, respectively. Intergenic circRNAs, intronic circRNAs, and antisense circRNAs accounted for 9.46, 2.24, and 4.01%, respectively (Figure 5E). Differential expression analysis revealed 212 (Figure 5A), 234 (Figure 5B), and 20 (Figure 5C) differentially expressed circRNAs in the LP vs. MP, LP vs. HP, and MP vs. HP comparison groups, respectively. Using a Venn diagram to highlight the DECs associated with each pair of samples (Figure 5D), it was found that there were 98 common DECs between the LP vs. HP and LP vs. MP comparison groups. Notably, among these DECs in the LP vs. HP and LP vs. MP comparison groups, bgrcirc_017953, bgrcirc_015858, and bgrcirc_003993 were simultaneously upregulated.

**Figure 6:**
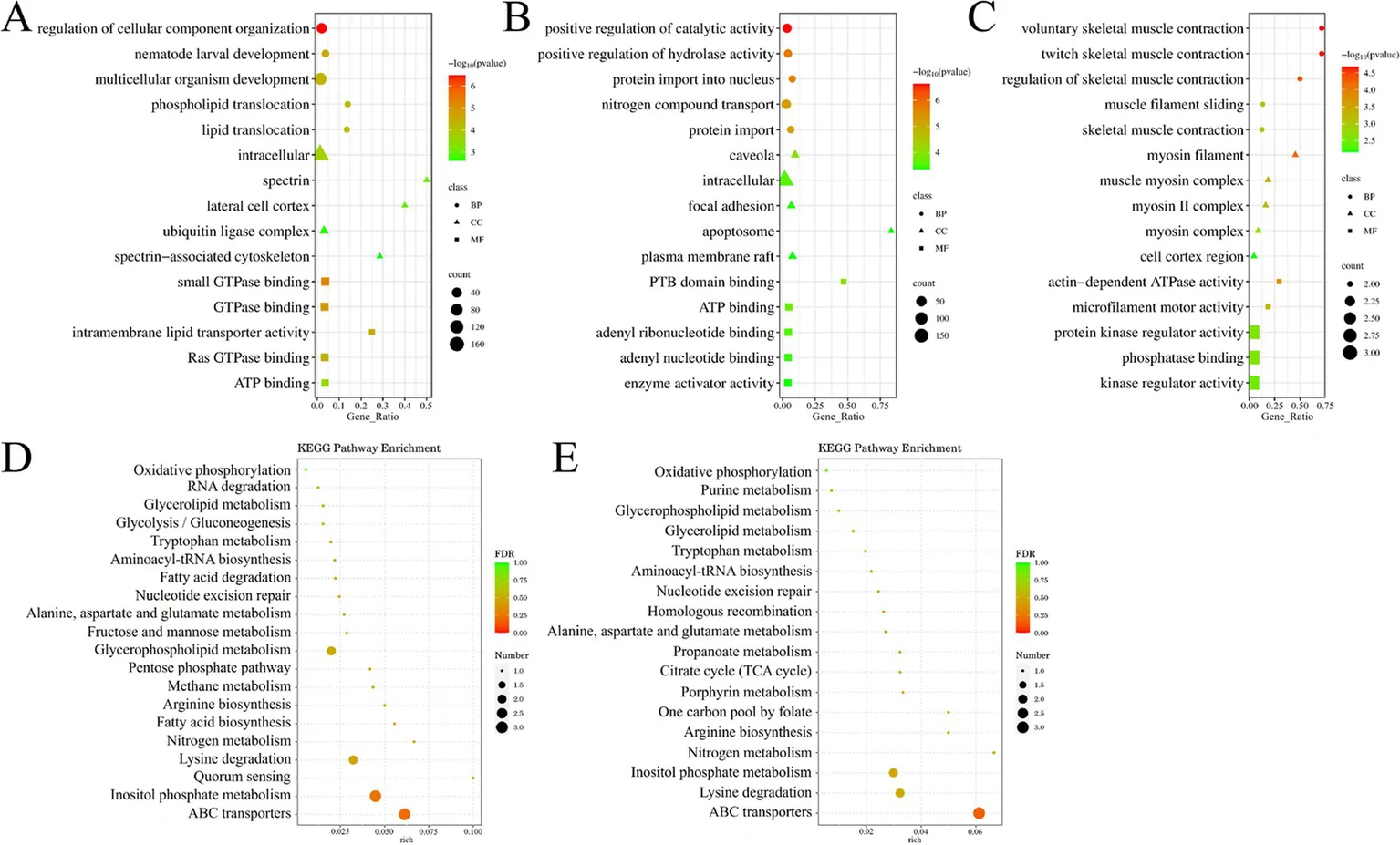
Functional enrichment analysis of DECs. GO enrichment analysis of LP vs. MP, LP vs. HP, and MP vs. HP **(A–C)**. KEGG enrichment analysis of LP vs. MP and LP vs. HP **(D,E)**. The general value range of FDR is 0–1, and the closer to zero, the more significant the enrichment.

The GO functional enrichment analysis revealed that DECs in the LP vs. MP group were significantly enriched in regulation of cellular component organization (Figure 6A and Supplementary Table S7), which has important implications for animal growth and development, including cell differentiation and organ development, tissue and organ function, cell proliferation and division, cell signaling, and cell metabolism and energy conversion (30). In the LP vs. HP group, the target genes of DECs were significantly enriched in positive regulation of catalytic activity and positive regulation of hydrolase activity (Figure 6B and Supplementary Table S7). Among these, positive regulation of catalytic activity plays a regulatory role in processes such as cell proliferation, differentiation, organ development, and cell migration during animal growth and development. However, the effects of positive regulation of hydrolase activity on animal growth and development are primarily manifested in nutrient digestion and absorption, activation of metabolic pathways, activation of growth factors, and degradation of waste products (31). These regulatory processes are crucial for normal animal growth and development and the maintenance of organ function. Additionally, the biological process entry regulation of cellular component organization was significantly enriched in the MP vs. HP group (Figure 6C and Supplementary Table S7), involving multiple biological processes and cellular components, and playing a vital role in maintaining normal muscle function and homeostasis. KEGG pathway analysis revealed that DECs in the LP vs. MP group were significantly enriched in the ABC transporters pathway (Figure 6D and Supplementary Table S7). The ABC transporter pathway plays a crucial role in animal growth and development, including nutrient absorption and metabolism, tissue development and organ formation, as well as drug metabolism and toxin clearance. These processes are essential for normal growth, development, and health in animals (32). Abnormal regulation of the ABC transporter pathway may lead to diseases such as growth and development abnormalities, metabolic disorders, and drug metabolism abnormalities. Additionally, DECs were significantly enriched in the inositol phosphate metabolism pathway, which is involved in various important biological processes within cells, including signal transduction, membrane phospholipid metabolism, and energy metabolism (33, 34). Notably, DECs in the LP vs. HP group were also significantly enriched in the ABC transporters pathway (Figure 6E and Supplementary Table S7). In contrast, the target genes of DECs in the MP vs. HP group were not enriched in any pathway.

### 3.6 Construction of the ceRNA coregulatory network

**Figure 7:**
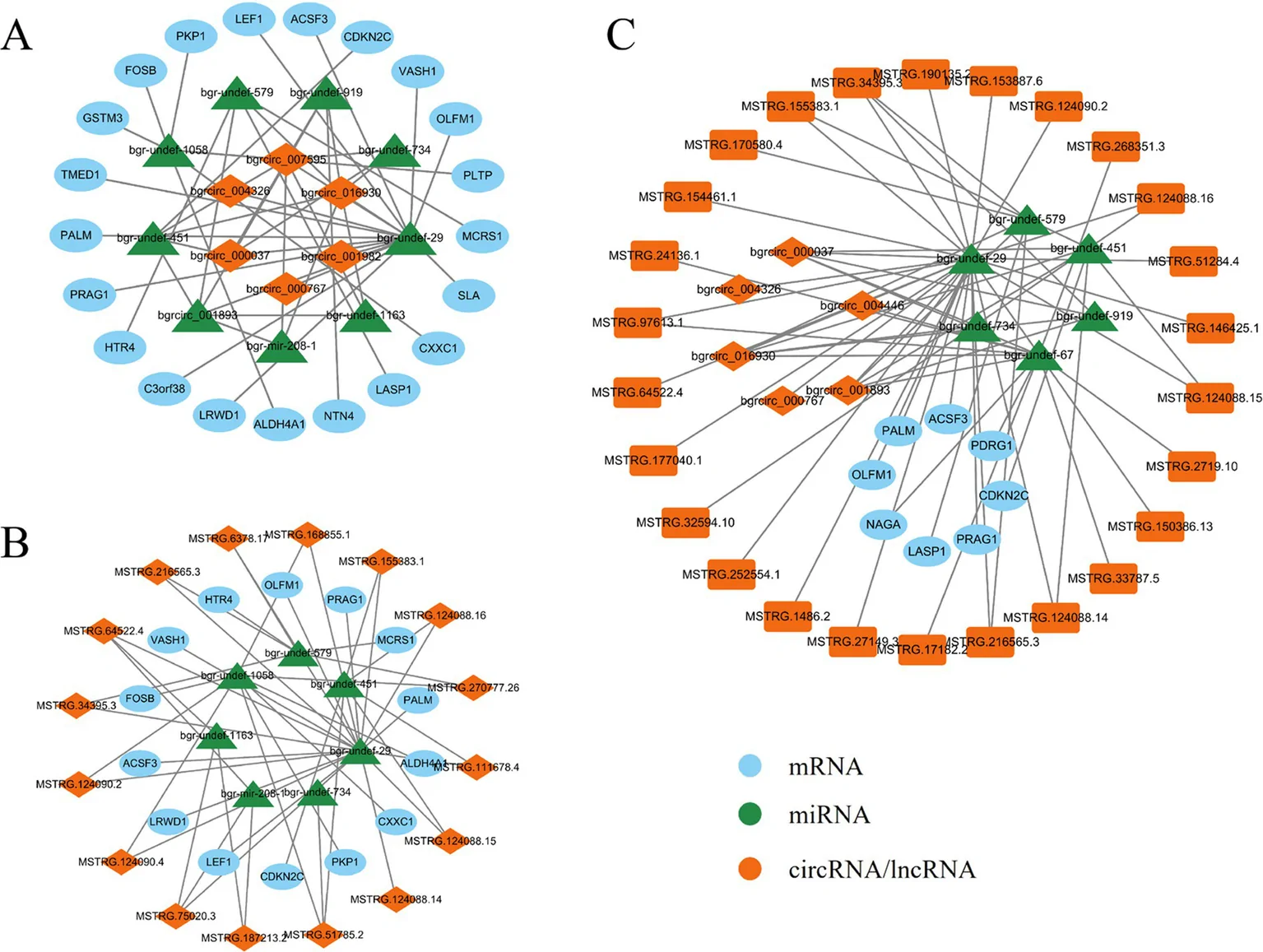
The ceRNA regulatory network. **(A)** CircRNA-miRNA-mRNA network of Jersey-yaks. **(B)** LncRNA-miRNA-mRNA network of Jersey-yaks. **(C)** LncRNA-circRNA-miRNA-mRNA network of Jersey-yaks. The colors indicate the mRNAs (blue) and miRNAs (green) lncRNAs/circRNAs (orange).

Based on the regulatory relationships among DEGs, DELs, DECs, and DEMs, we identified significantly differentially expressed lncRNAs, circRNAs, and mRNAs that are co-regulated by the same miRNAs (Supplementary Table S8). Network diagrams of circRNA-miRNA-mRNA and lncRNA-miRNA-mRNA were constructed based on the relationships among mRNAs, circRNAs, lncRNAs, and miRNAs (Figures 7A,B). The circRNA-miRNA-mRNA network primarily involves 6 circRNAs, 9 miRNAs, and mRNAs such as *PLTP*, *PRAG1*, and *CXXC1*. The lncRNA-miRNA-mRNA network primarily involves 7 miRNAs, 16 lncRNAs, and mRNAs such as *CDKN2C*, *LRWD1*, and *ACSF3*. To determine the regulatory relationships between coding and non-coding RNAs during the growth and development of Jersey-yak, we analyzed the regulatory relationships between differentially expressed RNAs through miRNA-miRNA, miRNA-circRNA, and miRNA-lncRNA. We identified significantly differentially expressed mRNAs, circRNAs, and lncRNAs co-regulated by miRNAs, thereby constructing a co-expressed lncRNA-circRNA-miRNA-mRNA network diagram between coding RNAs and non-coding RNAs (Figure 7C). This network diagram primarily includes 6 miRNAs, 6 circRNAs, 8 mRNAs, and multiple lncRNAs. MSTRG.1705804.4-bgrcirc_016930-bgr-undef-579-*LASP1*, MSTRG.24136.1-bgrcirc_004446-bgr-undef-67-*NAGA/PDRG1*, MSTRG.155383.1-bgrcirc_004326-bgr-undef-29-*ACSF3*, etc., may influence the growth and development of Jersey-yak.

### 3.7 Validation of RNA-seq results

**Figure 8:**
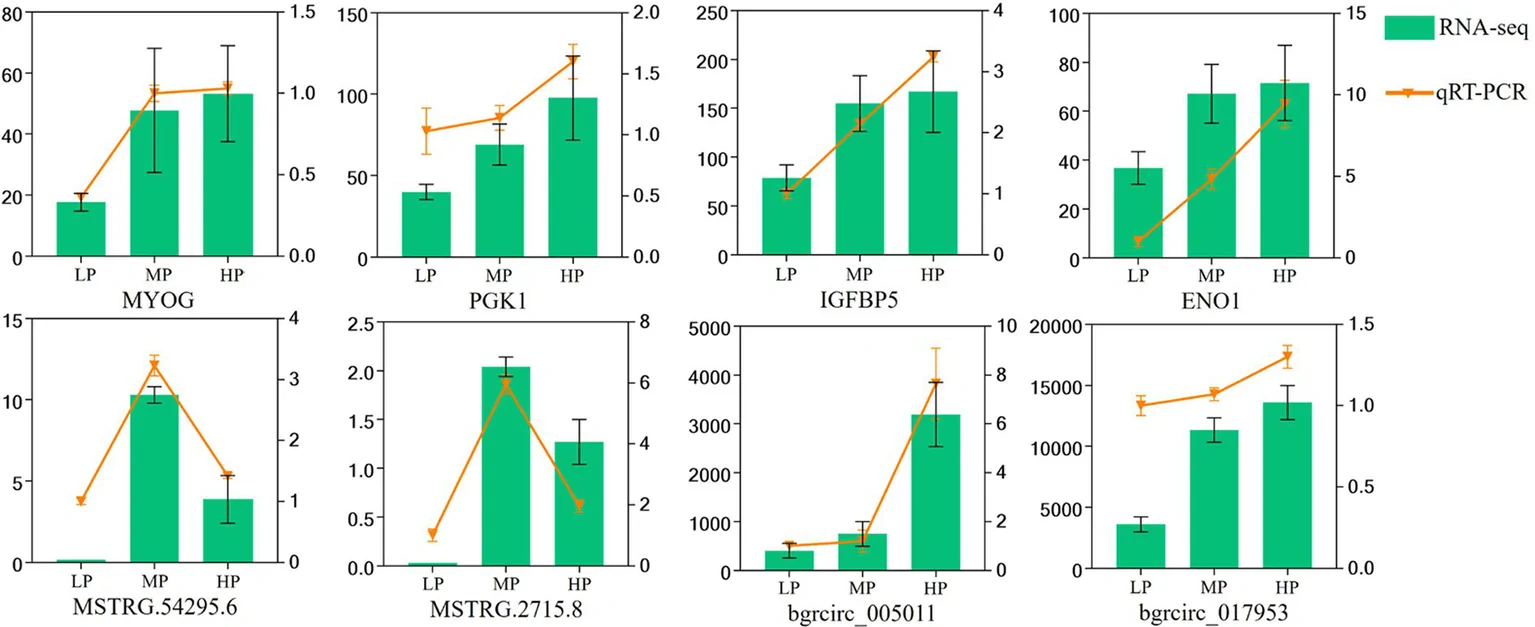
Validation of RNA-seq data using qPCR. The green bar chart represents RNA-seq data, while the yellow line chart represents qRT-PCR data.

Eight differentially expressed RNAs were randomly selected from the sequencing results of the Jersey-yak for qRT-PCR experiments, including 4 mRNAs (*MYOG*, *PGK1*, *IGFBP5*, and *ENO1*), 2 lncRNAs (MSTRG.2715.8 and MSTRG.54295.6), and 2 circRNAs (bgrcirc_005011 and bgrcirc_017953). The expression trends of the RNAs in the three comparison groups were found to be consistent with the qRT-PCR results (Figure 8 and Supplementary Table S9).

### 3.8 Correlation analysis of transcriptome and proteome expression regulation

**Figure 9:**
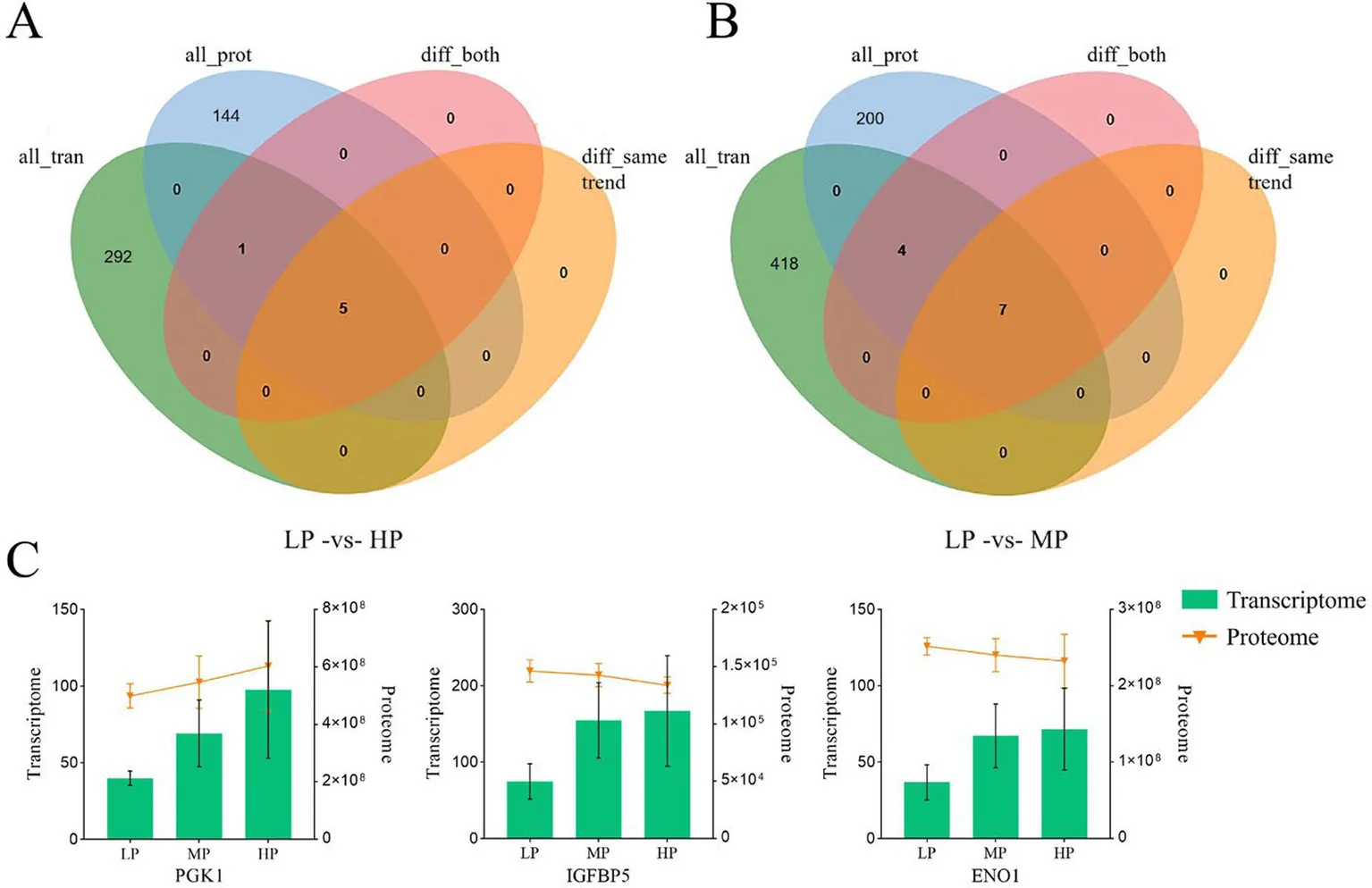
Comparison of differential analysis results and transcriptome between proteome. Venn diagrams of two comparison groups **(A,B)**. **(C)** Expression trends of *PGK1*, *IGFBP5*, and *ENO1* in the transcriptome and proteome.

Whole-transcriptome sequencing focuses on all RNA molecules in an organism across different tissues or species, including mRNA and non-coding RNA, while proteomics studies the expression, function, and regulatory mechanisms of all proteins in an organism. Correlating whole-transcriptome data with proteomic data allows us to establish connections from the transcriptional level of gene expression to the post-translational modifications and functional activities of proteins, thereby revealing a more comprehensive picture of the biological processes within an organism. Through the combined analysis of these two omics approaches, we can investigate the patterns of gene expression at different levels and uncover the regulatory relationships between them. However, the regulatory mechanisms, correlations, or expression trends between the transcriptome and the proteome are not always consistent. In fact, these inconsistencies in expression reflect the complexity and diversity of an organism’s biochemical state (35). The analysis revealed that in the LP vs. HP group, a total of 298 DEGs and 150 DEPs were identified. Six genes were differentially expressed in both the transcriptomic and proteomic analyses, five of which exhibited the same expression trend, including *LASP1*, *DUSP29*, *RNPEP*, *ENPP1*, and *SCN4B* (Figure 9A and Supplementary Table S10). In the LP vs. MP group, a total of 429 DEGs and 211 DEPs were identified. Eleven genes were differentially expressed in both the transcriptomic and proteomic analyses, of which 7 showed the same expression trend, including *PDLIM7*, *ATP2B2*, *CILP*, *HDGFL3*, *ENPP1*, *SCN4B*, and *KIF20A* (Figure 9B and Supplementary Table S10). However, no genes (or proteins) with common expression patterns were identified in the MP vs. HP group. To further verify the expression changes of *MYOG*, *PGK1*, *IGFBP5*, and *ENO1* at the protein level, we analyzed the proteomic data. The results showed that among the proteomic identifications, only *PGK1*, *IGFBP5*, and *ENO1* were identified and yielded quantitative data, whereas *MYOG* was not detected. Further comparison of the transcriptomic and proteomic results (Figure 9C) revealed that *PGK1* exhibited a consistent expression trend at both the transcript and protein levels. In contrast, although *IGFBP5* and *ENO1* were successfully identified in the proteome, their protein abundance trends were opposite to those observed in the transcriptomic sequencing results.

**Figure 10:**
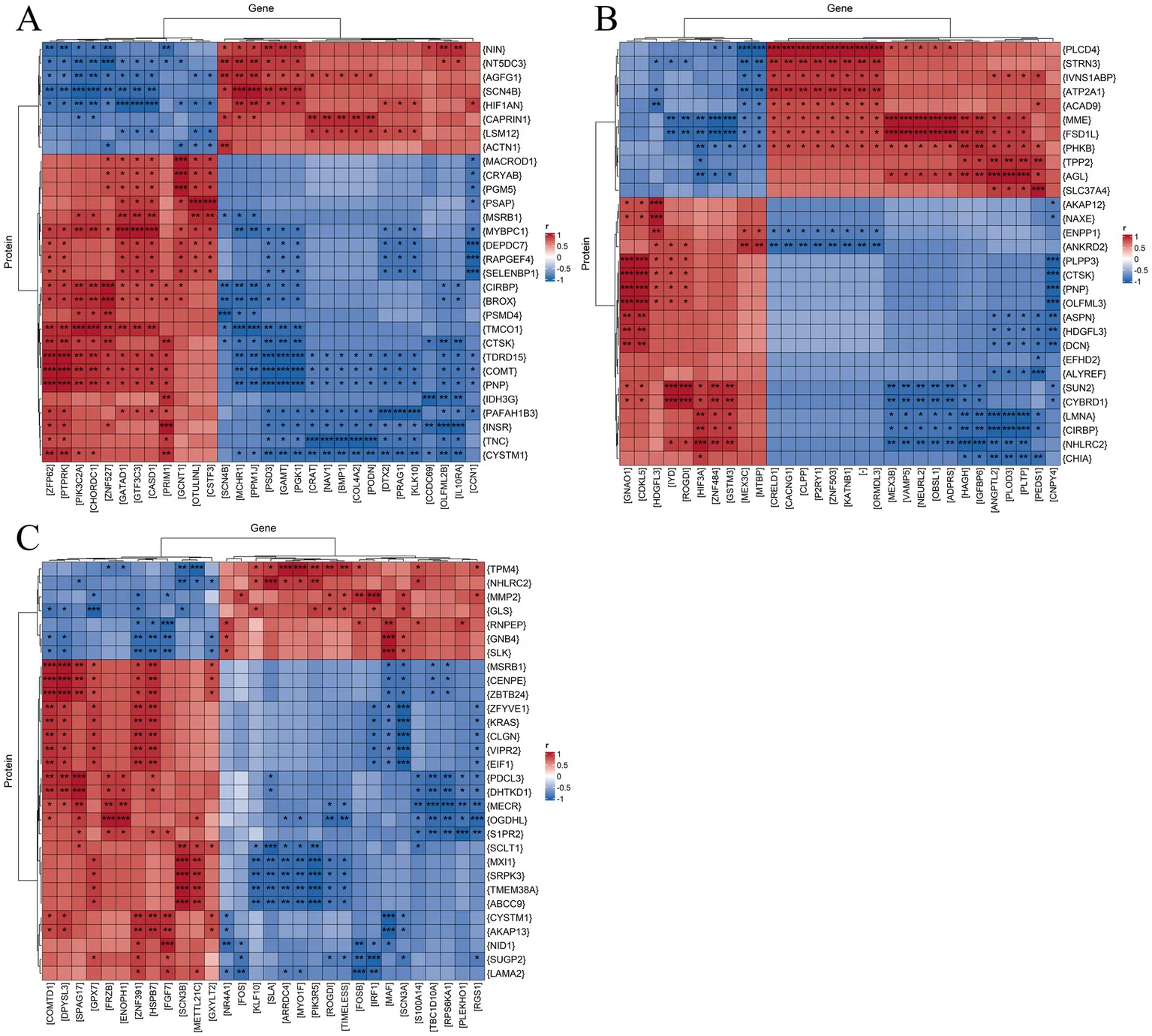
Comparison of differential analysis results and transcriptome between proteome. Transcriptome and proteome significant correlation hierarchical clustering heatmap. (**A**, **B**, **C**) are the correlation heatmaps for LP vs. HP, LP vs. MP, and MP vs. HP, respectively. *** in the small cells of the heatmap denotes correlation test *p* value < 0.001, ** denotes correlation test *p*-value < 0.01, * denotes correlation test *p* value < 0.05. Each column of the figure denotes a gene, and each row denotes a protein. Red: High expression; blue: Low expression.

To visually illustrate the similarities and differences in the correlations between the two omics elements, a Spearman correlation-based hierarchical clustering analysis was performed on the two omics elements, and the results were presented as a correlation-based hierarchical clustering heatmap. A heatmap of significant correlations was generated by selecting the top 30 differentially expressed genes (or proteins) ranked by their correlation coefficients (absolute values) (Figure 10). Analysis revealed that there are highly significant correlations between the expression levels of several genes and proteins; for example, there is a highly significant correlation between the gene *MYO1F* and the protein TPM4 in the MP vs. HP group. The gene *MYO1F* (Myosin IF) encodes a myosin protein, while the protein TPM4 (Tropomyosin 4) is encoded by the gene *TPM4*; these two genes and proteins may be related in terms of muscle-related cellular structure and function.

KEGG pathway enrichment analysis was performed on the genes (or proteins) identified through differential proteomics and transcriptomics analyses, and the results were plotted as a KEGG pathway enrichment diagram (Supplementary Figure S1). The analysis revealed that in the LPvsHP group, genes (or proteins) were jointly enriched in signaling pathways such as focal adhesions, Apelin, and HIF-1; in the LP vs. MP group, genes (or proteins) were commonly enriched in signaling pathways such as protein digestion and absorption, calcium, and TGF-beta; and in the MP vs. HP group, genes (or proteins) were commonly enriched in signaling pathways such as PI3K-Akt and ECM-receptor interactions.

## 4 Discussion

The growth and production performance of ruminants are affected by dietary protein levels. Studies have shown that adding crude protein to the diet can improve the production performance of beef cattle, and reducing or not adding crude protein will reduce the average daily gain of calves (36, 37). Protein in the diet is one of the primary sources of energy for animal tissues and plays a crucial role in the growth and development of ruminants. Additionally, the addition of appropriate amounts of protein to the diet significantly promotes the production performance and organ development of ruminants (38). Due to the prolonged dry season during the cold season in the Qinghai-Tibet Plateau region, there is a seasonal imbalance in forage supply. During the winter and spring seasons, Jersey-yak experience weight loss and reduced body condition due to insufficient forage, nutrient deficiencies in pasture grass, and harsh environmental conditions such as low oxygen levels and extreme cold, which can impair subsequent fattening and result in significant economic losses. Research findings indicate that supplementing early-weaned yak with feed of varying crude protein levels after grazing can improve calf growth performance while minimizing energy deficiency in mother yaks during the winter feed scarcity period (39). Researchers conducted supplementary feeding on 18-month-old yak during the cold season on the Qinghai-Tibet Plateau, and the results showed that the body weight and average daily weight gain of the supplementary feeding group were significantly improved (40). Research has shown that even a 2–3% difference in the protein content of the diet can have a significant impact on the growth and development of livestock (13). Therefore, this study used diets containing 15.16 and 17.90% crude protein to supplement the Jersey-yak. The experimental results showed that the average daily weight gain and total weight gain of the HP and MP groups were significantly higher than those of the control group, consistent with the aforementioned studies. These findings indicate that during the dry season, providing yak and their hybrid offspring with appropriate supplementary feeding, combined with a reasonable crude protein level in the diet, has significant potential to enhance their productivity and can appropriately reduce economic losses for herders.

Next, using whole-transcriptome sequencing technology, an average of 66,869,478, 64,964,700, and 67,520,923 clean reads were obtained from the LP, MP, and HP groups of Jersey-yak, respectively, with Q30 base distribution ranging from 93.69 to 94.18%. By aligning the reads to the reference genome, the alignment rates for each sample ranged from 93.57 to 94.47%. Differentially expressed RNAs were detected using qRT-PCR technology, and the results showed that the quantitative results of the selected RNAs were consistent with the sequencing results, indicating the reliability of the sequencing data. Through comparative analysis, 429, 298, and 84 differentially expressed mRNAs were identified in the LP vs. MP, LP vs. HP, and MP vs. HP comparison groups, respectively. Through GO and KEGG functional annotation analysis, these DEGs were mainly involved in pathways related to enzyme activity, metabolic processes, cell contraction, and tissue development. They were also enriched in pathways related to muscle growth and development, as well as fat cell storage and release, such as PI3K-Akt, MAPK, and regulation of fat cell lipolysis. Furthermore, these DEGs were enriched in pathways related to cellular adaptation to oxygen environments, such as the HIF-1 signaling pathway, which plays a key regulatory role in the body’s response to low oxygen concentrations or hypoxia and in maintaining oxygen homeostasis (41). This is consistent with the ability of Jersey-yak to adapt to low-oxygen environments in high-altitude regions. Through the Venn diagram, it was found that there were 74 DEGs co-expressed between LP vs. HP and LP vs. MP. The analysis found that these DEGs regulated the biological processes of adipocyte differentiation and proliferation, such as adipose tissue development and adipocyte differentiation. These DEGs were also found to be associated with glycosaminoglycan biosynthesis-heparin sulfate/heparin, PPAR signaling pathway, pantothenate and CoA biosynthesis (42, 43). Among them, glycosaminoglycan biosynthesis-heparin sulfate / heparin plays an important role in anticoagulation, cell signal transduction, extracellular matrix function and immune regulation *in vivo* (44). In addition, many of these co-expressed DEGs have been found to play an important role in regulating the growth and development of muscle tissue, including *COL15A1*, *FOXO1*, *PYGO2*, *SOX7*, *PLOD3*, and *NME3*, which are related to the development and regulation of muscle, bone and nervous system. The collagen type XV alpha 1 chain (*COL15A1*) originally belonged to non-fibrous collagen and is present in many tissues (45). Studies on neonatal heart, skeletal muscle, and smooth muscle tissues have shown that muscle RNA has high levels of type XV collagen mRNA expression, indicating that it is not only localized in fibroblasts in the endothelium but also in myoblasts. Interestingly, certain epithelial cells in the kidney, lung, pancreas, and placenta can also synthesize XV-type collagen mRNA. These findings suggest that XV-type collagen transcripts are widely distributed, with the primary producers being mesenchymal-derived cells, particularly muscle cells and fibroblasts (46). *COL15A1* encodes the *α* chain of collagen XV, primarily produced by mesenchymal cells, including muscle cells, adipocytes, fibroblasts, and neurons (47). Therefore, the *COL15A1* gene plays a crucial role in the growth and development of Jersey-yak and the formation of tissue structure. The forkhead box protein O1 (*FOXO1*) gene encodes the FoxO1 protein, which is a member of the FoxO protein family and plays a crucial regulatory role in various cellular processes. Studies in cattle have revealed that it exerts its functions by binding to the promoters of downstream target genes or interacting with other transcription factor proteins (48). Studies have shown that the *FOXO1* gene is highly expressed in insulin-responsive tissues, including the pancreas, liver, skeletal muscle, adipose tissue, and bone, and plays a crucial role in regulating muscle growth, metabolism, cell proliferation, and differentiation (49). Its specific function involves inhibiting the downstream key factor Akt1/2 of the insulin signaling pathway, thereby impairing skeletal muscle development. However, studies in mice have found that knocking out *FOXO1* can restore the developmental capacity of skeletal muscle and other tissues (50). Additionally, studies have shown that certain non-coding RNAs can regulate *FOXO1* expression, thereby influencing muscle cell proliferation and differentiation (51). These findings suggest that reducing *FOXO1* gene expression may promote muscle cell generation, thereby facilitating muscle growth and development. Notably, *FOXO1* gene expression was downregulated in both the LP vs. HP and LP vs. MP comparison groups, consistent with its functional role. Additionally, genes such as *SOD3*, *LASP1*, and *ACAD8*, which are associated with the development of muscle, bone, and organs in Jersey-yak, were differentially expressed and upregulated in both supplementation groups (52, 53).

Although an increasing number of researchers are beginning to study the mechanisms related to lncRNA, the functions of lncRNA remain largely unknown. However, researchers believe that lncRNA is involved in many fundamental cellular processes (54), and the half-life of nuclear lncRNA is very short (<2 h), making it highly susceptible to regulatory functions (55). During biological development, lncRNA, as a non-coding RNA, acts as an important regulatory factor in gene expression during the processing of genetic information in living cells and interacts with major cellular pathways such as proliferation, differentiation, and apoptosis (56). In this study, 356, 394, and 302 differentially expressed lncRNAs were identified in the three comparison groups of LP vs. HP, LP vs. MP and MP vs. HP, respectively. Through the Venn diagram, 73 lncRNAs were found to be differentially co-expressed between the two control groups and the supplementary feeding group. The number of exons of most lncRNAs identified in this study is 2–3, and the length of most lncRNA exons is about 200 bp, which is basically consistent with the characteristics of lncRNAs identified in other mammals (57, 58). GO and KEGG functional enrichment analysis showed that the target genes of these DELs are mainly involved in biological processes related to enzyme activity, cell proliferation and differentiation, and metabolism. Among these, the *FGF2* gene is a key regulator of muscle cell proliferation and differentiation. Therefore, lncRNAs such as MSTRG.76433, MSTRG.76436, MSTRG.76443, and MSTRG.76478 can be studied as co-expressed lncRNAs of the *FGF2* gene. From an evolutionary perspective, the Wnt signaling pathway is highly conserved and plays a significant role in embryonic development, growth and development, and the regeneration of adult tissues (59). It is also crucial for maintaining genetic stability and plays a key role in determining cell fate, differentiation, apoptosis, cell movement, and stem cell maintenance (60). In this study, 14 target mRNAs of differentially expressed lncRNAs in the LP vs. MP group were significantly enriched in the Wnt signaling pathway, among which MSTRG.239143.1, which regulates the *WNT3A* gene, was upregulated in the MP group. The *WNT3A* gene encodes the Wnt-3a protein, which is an important member of the Wnt signaling pathway. Studies have shown that the Wnt-3a protein participates in regulating the proliferation, differentiation, and formation of skeletal cells, and the absence or mutation of *WNT3A* may lead to skeletal developmental abnormalities or skeletal diseases (61). Previous studies have shown that Wnt3a can regulate the functions of *MYOD* and *MYOG* (62), which are highly expressed genes in fast-twitch and slow-twitch muscle fibers, respectively. Overexpression of *WNT3A* promotes the expression of myosin heavy chain type I (*MyHC-I*) in slow-twitch skeletal muscle fibers and facilitates cell differentiation toward slow-twitch fibers (63).

As research has progressed, it has been discovered that circRNAs are indispensable in muscle growth and development. circRNAs are a novel type of circular endogenous RNA formed by reverse splicing of mRNA precursors. Due to their circular closed-loop structure, they exhibit conservation and stability, unlike linear RNAs, which lack a 5′ cap and 3′ poly(A) tail (64). In recent years, numerous studies have shown that circRNAs can regulate gene expression through various mechanisms, including acting as “molecular sponges” to absorb small RNA molecules or translating proteins to regulate muscle growth (65, 66). Recent research has further revealed that circRNAs can also regulate muscle growth and development by interacting with RNA-binding proteins (RBPs) (67). Research has found that CDR1as (the antisense transcript of cerebellar degeneration-related protein 1, also known as ciRS-7, is a neuronal circRNA that plays a crucial role in regulating muscle differentiation) is highly expressed in the muscles of mid-embryonic goat fetuses and is activated during the initial stages of *in vitro* muscle differentiation. *MyoD* promotes CDR1as by binding to its 5′ end region. Overexpression or knockout of CDR1as significantly induces or inhibits muscle differentiation, respectively. By competitively binding to miR-7, CDR1as alleviates miR-7-mediated downregulation of *IGF1R*, thereby activating muscle differentiation. These findings highlight the critical role of CDR1as in muscle differentiation (66). GO analysis revealed that the target genes of these DECs are involved in enzyme activity regulation, cell proliferation, differentiation, and muscle contraction. Among them, the MYL1 protein encoded by myosin light chain 1 (*MYL1*) participates in muscle fiber assembly and muscle development, promoting muscle cell growth and differentiation, and plays an important role in muscle growth and development. The bgrcirc_017953 targeting the *MYL1* gene was upregulated in both the LP vs. HP and LP vs. MP groups, suggesting that bgrcirc_017953 may have a regulatory role in muscle development in Jersey-yak. Through KEGG pathway enrichment analysis, it was found that the target genes of DECs in the LP vs. HP and LP vs. MP groups were significantly enriched in the ABC transport pathway. The ABC transport pathway plays an important regulatory role in animal growth and organ development, with the *ABCC1*, *ABCA1*, and *ABCG2* genes all enriched in this pathway. These genes are involved in the transport and metabolic regulation of substances, influencing animal growth and development processes. The expression of bgrcirc_011298 related to *ABCC1* gene was downregulated in both HP and MP groups, and there may be competitive regulation between them (68). Its function research deserves further study. One limitation of the present study is that the identified circRNAs were not further validated by divergent primer PCR and Sanger sequencing across the back-splice junctions. Therefore, the circRNAs identified in this study and the corresponding ceRNA regulatory network should be considered as bioinformatically predicted candidates rather than experimentally confirmed regulatory interactions. Future studies will focus on validating the back-splice junctions of key circRNAs and investigating their biological functions through molecular experiments.

ceRNA is a regulatory mechanism in living organisms involving mRNA, lncRNA, miRNA, and circRNA. These RNA molecules regulate each other by competitively binding to common miRNAs, forming a complex regulatory network. The fundamental principle of this mechanism is that RNA molecules can compete for miRNA molecules by possessing identical or similar miRNA response elements (MREs), thereby influencing each other’s expression and function (69, 70). This interaction forms the ceRNA network, which plays a role in many biological processes such as cell proliferation, differentiation, and development. In order to determine the regulatory relationship between coding RNAs and non-coding RNAs in the growth and development of Jersey-yak, based on the regulatory relationship between differentially expressed RNAs of miRNA-mRNA, miRNA-circRNA and miRNA-lncRNA. We identified significantly differentially expressed mRNAs, circRNAs and lncRNAs co-regulated by miRNAs, thus constructing a ceRNA regulatory network diagram between coding RNAs and non-coding RNAs. The ceRNA network contains 8 DEGs, 6 DECs, 6 DEMs, and multiple DELs. This study showed that MSTRG.1705804.4-bgrcirc_016930-bgr-undef-579-*LASP1*, MSTRG.24136.1-bgrcirc_004446-bgr-undef-67-*NAGA*/*PDRG1*, MSTRG.155383.1-bgrcirc_004326-bgr-undef-29-*ACSF3* may be involved in the regulation of growth and development of Jersey-yak. Among them, bgr-undf-29 targeted multiple DELs such as MSTRG.155383.1, MSTRG.190135.23 and MSTRG.51284.4, as well as 4 DEGs such as *OLFM1*, *PRAG1*, *ACSF3* and *PALM*. Olfactomedin 1 (*OLFM1*), also known as “noelin” or “pancortin,” is a secreted glycoprotein that has garnered significant attention in recent years (71). This protein is highly expressed in brain tissue, particularly in regions closely associated with cognitive processes, such as the hippocampus and cortex (72). Research has shown that the *OLFM1* gene has an inhibitory effect on osteoclast formation in mouse cells (73). Acyl-CoA synthetase family member 3 (*ACSF3*) encodes an enzyme in the acyl-CoA synthetase family, which activates fatty acids by catalyzing the formation of a thioester bond between fatty acids and coenzyme A (74). Research has shown that *ACSF3* is associated with meat traits, carrying quantitative trait loci related to beef production traits. Functional analysis revealed its involvement in biologically relevant processes such as fatty acid oxidation, biosynthesis, and lipid storage (75). The results indicate that the genes in this ceRNA network, along with their corresponding miRNAs, lncRNAs, and circRNAs, may influence the growth and development of skeletal muscle and fat formation in Jersey-yak. Furthermore, the ceRNA network constructed in this study is primarily based on bioinformatics predictions and expression correlation analyses; its results should be regarded as candidate regulatory relationships. Future work will need to further validate these regulatory relationships by combining analyses of negative correlations between miRNAs and target RNAs, positive correlations between competing RNAs, multiple-test corrections, and validation through molecular experiments.

Analysis revealed that there were 5 differentially expressed genes (or proteins) with identical expression trends in the LP vs. HP group, and 7 such genes (or proteins) in the LP vs. MP group. Among these, LIM and SH3 protein 1 (*LASP1*) is a small multidomain actin-binding protein associated with the stabilization of actin filaments (52, 76). Studies have shown that *LASP1* is a candidate gene for body height in animals, as its expression levels influence cartilage formation, osteogenic differentiation, and cell migration. The protein-binding pathways in which it participates play a crucial role in animal growth and development; its upregulation in the LPvsHP group may be associated with cartilage formation in Jersey-yak. In this study, *LASP1* exhibited significant changes at the transcriptional level, while changes at the protein level were relatively limited, suggesting that the expression of this gene in the skeletal muscle of Jersey-yak may be influenced by post-transcriptional regulatory mechanisms. As an important structural protein involved in cytoskeletal organization and the formation of adhesion complexes, the function of *LASP1* depends not only on its expression level but also on protein localization, interaction networks, and stability regulation (77). When skeletal muscle is subjected to nutritional and mechanical stimuli, cytoskeletal-associated proteins participate in muscle adaptation processes by integrating mechanical and metabolic signals. Therefore, the partial discrepancy between *LASP1* expression at the RNA and protein levels may reflect the fine-tuned regulation of dietary-induced protein levels in the skeletal muscle tissue of Jersey-yak, rather than a simple transcriptional response. Ectonucleotide pyrophosphatase/phosphodiesterase 1 (*ENPP1*) is a transmembrane glycoprotein that was first identified as a negative regulator of bone mineralization. The *ENPP1* gene hydrolyzes ATP into AMP and pyrophosphate, thereby inhibiting hydroxyapatite deposition and negatively regulating bone mineralization (78). Pyrophosphate is a potent mineralization inhibitor capable of effectively slowing or inhibiting the deposition of calcium salts (primarily hydroxyapatite) in soft tissues and bones (79). As pyrophosphate concentrations increase, its inhibitory effect on hydroxyapatite formation also intensifies. Therefore, increased expression of the *ENPP1* gene leads to a slowing or inhibition of the bone mineralization process, which may result in osteomalacia and adversely affect skeletal health and development. It is worth noting that the *ENPP1* gene (or protein) was differentially downregulated in both the LPvsHP and LPvsMP groups, which may be related to the regulation of skeletal development in Jersey-yak. Studies have found that *ENPP1* is expressed in various tissues and can regulate neural, immune, hormonal, musculoskeletal, and cardiovascular functions in mammals (80). Analysis of the correlation between transcriptomic and proteomic expression revealed highly significant correlations between the expression of multiple genes and proteins, such as the gene *MYO1F* and the protein TPM4, the gene *GTF3C3* and the protein MYBPC1, and the gene *ZFP62* and the protein TDRD15. The relationships between genes and proteins are diverse and complex, and indirect interaction pathways may also exist, such as shared participation in the same signaling pathway or interactions with other intermediary proteins. However, further research is needed to elucidate exactly how genes and proteins interact and function within the Jersey-yak.

Notably, no significant transcriptome–proteome concordance was observed between the MP and HP groups in this study, which may reflect an adaptive regulation of skeletal muscle in response to high dietary protein supply rather than a simple insufficiency of molecular responsiveness. One possible explanation is that, once dietary protein levels reach the range sufficient to meet muscle growth requirements, the capacity for skeletal muscle protein synthesis may progressively approach its physiological upper limit, manifesting as a certain degree of “saturation effect.” Previous studies have shown that muscle protein synthesis in animals exhibits a dose-dependent response to protein intake; however, when protein supply exceeds the level required for maximal deposition, further increases in protein input fail to sustainably promote muscle protein accretion, and instead are predominantly associated with reduced nitrogen utilization efficiency and adjustments in protein turnover (81, 82). Consequently, despite the nutritional differences between the MP and HP groups, their final protein compositions may tend to converge, thereby accounting for the lack of clear correspondence between transcriptional changes and protein alterations.

Although the ceRNA network provides important clues for interpreting the regulatory relationships among mRNA, lncRNA, circRNA, and miRNA, it still has certain limitations. First, the construction of the ceRNA network in this study relied primarily on bioinformatics predictions and differential expression analysis; however, miRNA target prediction algorithms inherently have a certain false-positive rate, which may lead to inaccuracies in some of the identified interactions. Furthermore, this study only performed qRT-PCR experiments on the screened differentially expressed RNAs and did not conduct functional validation at the gene knockdown/overexpression or protein levels; the exact roles of key differentially expressed genes (such as *MYOG*, *PGK1*, *IGFBP5*, and *ENO1*) still require confirmation in subsequent experiments. It is worth noting that the sample size of only three biological replicates per group limits statistical power and may reduce the sensitivity for detecting genes with subtle expression differences. To enhance the robustness of the findings, all RNA samples were collected under identical experimental conditions and exhibited high sequencing quality. Therefore, the current results should be regarded as exploratory hypotheses and need to be validated in larger, independent cohorts to more comprehensively elucidate the molecular mechanisms by which dietary protein levels regulate skeletal muscle development in Jersey-yak.

## 5 Conclusion

In this study, whole-transcriptome sequencing was performed on Jersey-yak fed diets with varying protein levels. Bioinformatics analysis identified numerous differentially expressed coding RNAs and non-coding RNAs, which play crucial roles in the skeletal muscle growth and development of Jersey-yak. Correlation analysis identified several genes co-expressed in both the transcriptome and proteome, such as *LASP1*, *DUSP29*, *PDLIM7*, and *ATP2B2*. These genes are associated with cell proliferation, differentiation, and muscle growth and development in Jersey-yak. This study provides a theoretical foundation for understanding the mechanisms underlying skeletal muscle development in large livestock.

## Data Availability

RNA-seq data from this study can be read from NCBI sequences (Accession Number: PRJNA1310280).

## Footnotes

1
https://github.com/marvin-jens/find_circ/

2
http://www.mirbase.org/

3
http://www.geneontology.org

4
https://www.genome.jp/kegg/

## Supplementary material

SUPPLEMENTARY TABLE S1 Composition and nutrient level of supplementary diet.

SUPPLEMENTARY TABLE S2 Summary of sequencing reads and mapping to the reference genome.

SUPPLEMENTARY TABLE S3 Identified RNAs.

SUPPLEMENTARY TABLE S4 List of differentially expressed RNAs.

SUPPLEMENTARY TABLE S5 Functional analysis of differentially expressed RNAs.

SUPPLEMENTARY TABLE S6 LncRNA cis-prediction of target gene information.

SUPPLEMENTARY TABLE S7 Functional analysis of differentially expressed miRNAs.

SUPPLEMENTARY TABLE S8 ceRNA abstract.

SUPPLEMENTARY TABLE S9 qRT-PCR primer information and RNA expression levels.

SUPPLEMENTARY TABLE S10 Correlation analysis.
