# Supplementary material for: Comprehensive analysis of differential mRNA, lncRNA and circRNA and their ceRNA network in the Longissimus Dorsi Muscle of Jerseyyak in response to dietary protein levels
Source: Front Vet Sci. 2026 Aug 19;13:1924842. doi: 10.3389/fvets.2026.1924842 (PMC13533735; doi:10.3389/fvets.2026.1924842)
Supplement: Supplementary file 1 [file Image_1.pdf]

Whole transcriptomic analysis revealed the effects of different dietary protein levels on the growth and development of Jersey-yak

Additional Figure

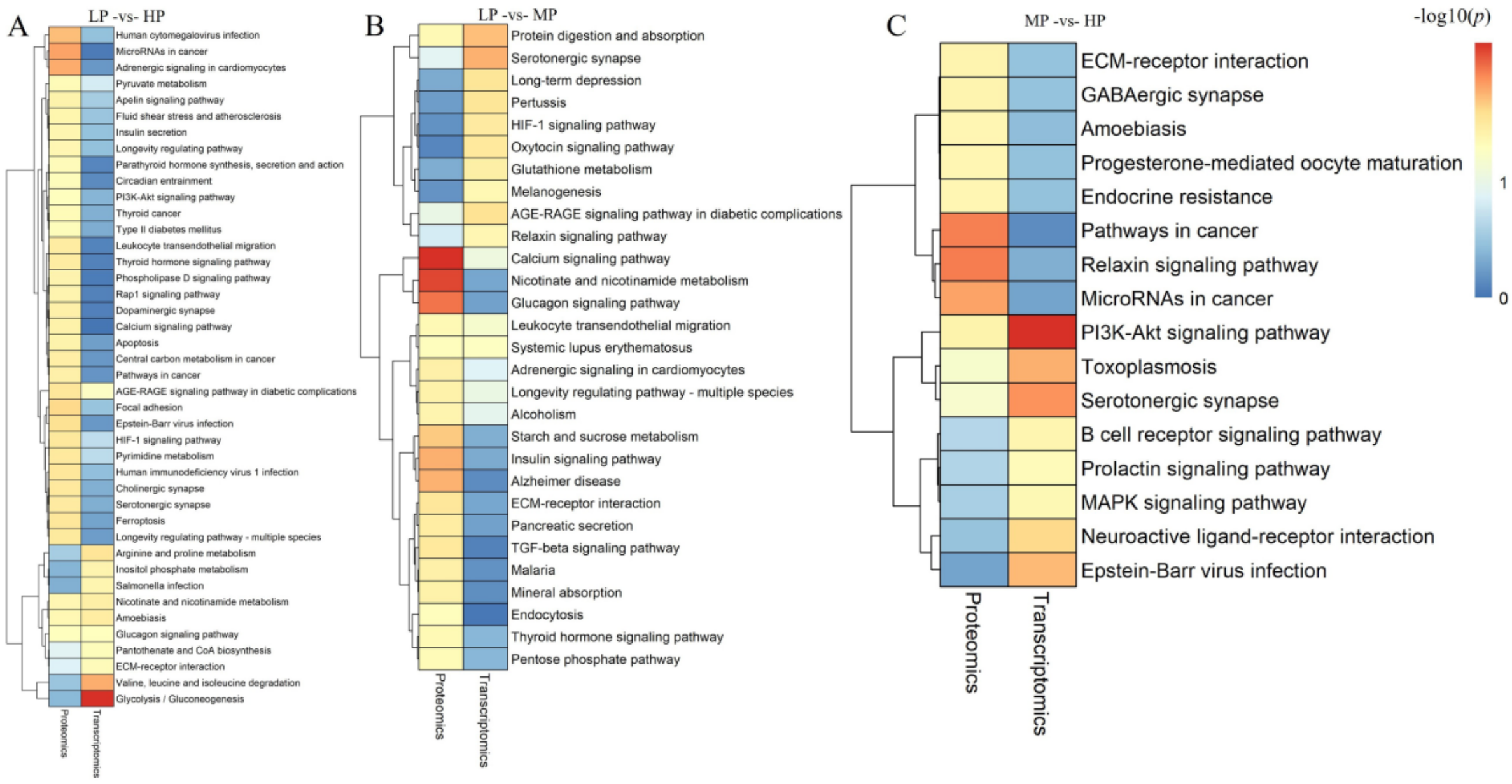

Figure S1. Heatmaps showing KEGG enrichment analysis of proteomics or transcriptomics data across the three comparison groups. A: LP vs. HP; B: LP vs. MP; C: MP vs. HP.
